# Feasibility of Telemedical HINTS (Head Impulse-Nystagmus-Test of Skew) Evaluation in Patients With Acute Dizziness or Vertigo in the Emergency Department of Primary Care Hospitals

**DOI:** 10.3389/fneur.2021.768460

**Published:** 2022-02-11

**Authors:** Rascha von Martial, Christina Leinweber, Nikolai Hubert, Holger Rambold, Roman Ludwig Haberl, Gordian Jan Hubert, Peter Müller-Barna

**Affiliations:** ^1^Department of Neurology, TEMPiS Telestroke Center, Academic Teaching Hospital of the Ludwig-Maximilians-University, München Klinik, Munich, Germany; ^2^Department of Neurology, InnKlinikum gKU Altötting und Mühldorf, InnKlinikum Altötting, Altötting, Germany; ^3^Department of Neurology, University of Regensburg, Regensburg, Germany; ^4^Department of Neurology, MVZ Kliniken Mühldorf, Mühldorf am Inn, Germany

**Keywords:** dizziness, vertigo, telemedicine, emergency department, stroke, HINTS, video head impulse test

## Abstract

**Background:**

Acute dizziness, vertigo and imbalance are common symptoms in emergency departments. Stroke needs to be distinguished from vestibular diseases. A battery of three clinical bedside tests (HINTS: Head Impulse Test, Nystagmus, Test of Skew) has been shown to detect stroke as underlying cause with high reliability, but implementation is challenging in primary care hospitals. Aim of this study is to prove the feasibility of a telemedical HINTS examination *via* a remotely controlled videooculography (VOG) system.

**Methods:**

The existing video system of our telestroke network TEMPiS (Telemedic Project for Integrative Stroke Care) was expanded through a VOG system. This feature enables the remote teleneurologist to assess a telemedical HINTS examination based on inspection of eye movements and quantitative video head impulse test (vHIT) evaluation. ED doctors in 11 spoke hospitals were trained in performing vHIT, nystagmus detection and alternating cover test. Patients with first time acute dizziness, vertigo or imbalance, whether ongoing or resolved, presented to the teleneurologist were included in the analysis, as long as no focal neurological deficit according to the standard teleneurological examination or obvious internal medicine cause was present and a fully trained team was available. Primary outcome was defined as the feasibility of the telemedical HINTS examination.

**Results:**

From 01.06.2019 to 31.03.2020, 81 consecutive patients were included. In 72 (88.9%) cases the telemedical HINTS examination was performed. The complete telemedical HINTS examination was feasible in 46 cases (63.9%), nystagmus detection in all cases (100%) and alternating covert test in 70 cases (97.2%). The vHIT was recorded and interpretable in 47 cases (65.3%). Results of the examination with the VOG system yielded clear results in 21 cases (45.7%) with 14 central and 7 peripheral lesions. The main reason for incomplete examination was the insufficient generation of head impulses.

**Conclusion:**

In our analysis the telemedical HINTS examination within a telestroke network was feasible in two thirds of the patients. This offers the opportunity to improve specific diagnostics and therapy for patients with acute dizziness and vertigo even in primary care hospitals. Improved training for spoke hospital staff is needed to further increase the feasibility of vHIT.

## Introduction

Vertigo and dizziness are among the most common symptoms in the emergency department (ED) with about 4% of emergency patients suffering from it (1). Lifetime prevalence of medium and intense vertigo and dizziness is about 30% (2). About 4.4 million annual visits to emergency rooms in the United States of America are due to vertigo and dizziness (3). A relevant portion of patients with dizziness and vertigo are misdiagnosed in the ED (4). In particular, failing to identify stroke as a cause of dizziness/vertigo has fatal consequences (5). According to recent estimates about 4% to 10% of ED patients with dizziness and vertigo as leading symptoms have a stroke (6, 7) and misdiagnosis of peripheral vestibular failure is estimated to account for up to 60% of stroke cases during the initial classification of patients in the ED (5, 8, 9).

The quick differentiation between a central and a peripheral cause of dizziness/vertigo is crucial to start appropriate therapy in time, e.g., stroke unit therapy including thrombolysis in selected cases. In order to differentiate central and peripheral causes, the HINTS (Head Impulse test, Nystagmus, Test of Skew) is the best known clinical test battery in cases of acute dizziness/vertigo (10). The HINTS examination is a battery of bedside clinical tests and consists of three examinations: the head impulse test, characterization of nystagmus and test of skew. It has been shown to be more reliable than a cranial MRI (Magnetic Resonance Imaging) in the first 48 h after the onset of vertigo symptoms in patients with acute vestibular syndrome (AVS) (11). Within the HINTS examination, the single best predictor for stroke is the clinically assessed horizontal head impulse test (HIT). A bilaterally normal result increases the odds of a stroke and an abnormal HIT is predictive for a peripheral vestibulopathy (9). HIT is also the most challenging to perform and to interpret, and the expertise of the examiner affects results with sensitivity of maximum 70% [compared to video head impulse test (vHIT)] even in experienced neuro-otologists (12, 13). This is, amongst other reasons, due to the fact that in a relevant proportion of individuals early corrective saccades in eye movements or so-called covert saccades are clinically not detectable. Clinically visible saccades with later onset are referred to as overt saccades. The clinical differentiation of correcting saccades from Nystagmus might therefore be difficult (14). Furthermore, HIT is a subjective test without an objective verifiable approach. The quantitative analysis of HIT, called vHIT overcomes these limitations (10, 15–17). It has already been shown in various studies that, compared to clinical bedside HIT, vHIT improves the HINTS examination (10, 17). Moreover, HINTS examination's sensitivity and specificity are higher when tests are performed and interpreted by a trained neurologist rather than by emergency physicians (18). Those specialists are mostly not available in the ED of primary care hospitals and due to lack of expertise HINTS examinations are often not performed at all (9). In our study we built on those experiences and performed HINTS examinations *via* a VOG system. The technical examinations were included in a standard videoconferencing system to be assessed remotely by an experienced teleneurologist. Our telemedicine-supported project to examine patients with dizziness and vertigo (called TeleVertigo) may offer improved acute care in patients with acute dizziness, vertigo or imbalance in EDs of primary care hospitals (19). This study aims to analyze the feasibility of a telemedical HINTS examination.

## Materials and Methods

This study was based on our telemedical stroke network cohort. The Telemedic Project for Integrative Stroke Care (TEMPiS) is a telestroke unit network in South East Bavaria, Germany, with two comprehensive stroke centers (hubs) and 24 primary stroke centers (spokes) (20). Within this network any patient with suspicion of acute stroke admitted to the ED in one of the spokes is presented to a teleneurologist provided there is no consultant neurologist on site. In addition to the evaluation of computed tomography (CT) scans (or MRI) hyperacute treatment, e.g., recanalization therapies and stroke unit care, can be recommended. In the network more than 7.000 teleconsultations are performed annually.

Since November 2018, the TeleVertigo project has been implemented on the already existing structure of the TEMPiS network in 11 spokes so that any patients presented within the TEMPiS network could be telemedically examined concerning dizziness and vertigo (19). A VOG system plugged into the existing video conferencing system was established. This feature enables the remote teleneurologist to see even subtle eye movements and to instruct and evaluate the vHIT. According to the standard TEMPiS procedure, any patient examined *via* videoconference first undergoes medical history screening (including pre-existing conditions and medication) and stroke assessment including National Institute of Health Stroke Scale (NIHSS) classification. In dizzy and vertiginous patients and those with acute balance disturbance a more detailed neurological examination is performed provided it is possible *via* standard camera. In this study, according to a consensus statement of the Barany Society, we defined dizziness as “the sensation of disturbed or impaired spatial orientation without a false or distorted sense of motion” and vertigo as “the sensation of self-motion when no self-motion is occurring or the sensation of distorted self-motion during an otherwise normal head movement” (21). If there are clear signs of either a central origin (mostly concomitant focal neurological deficits) or a primary internal disease in history, clinical examination, laboratory results or imaging explaining the patients' symptoms, they are admitted either to the stroke unit or to internal medicine ward respectively. If standard teleneurological examinations reveal no conclusive signs concerning the symptoms' etiology, examination with the VOG system is added. An algorithm for the HINTS examination for dizzy/vertiginous patients aimed at differentiating peripheral vs. central origin was implemented. Further detail can be found in an already published account on this system (19). This study exclusively evaluates the telemedical HINTS examination.

### Standard Operating Procedures and Teaching

**Figure 1:**
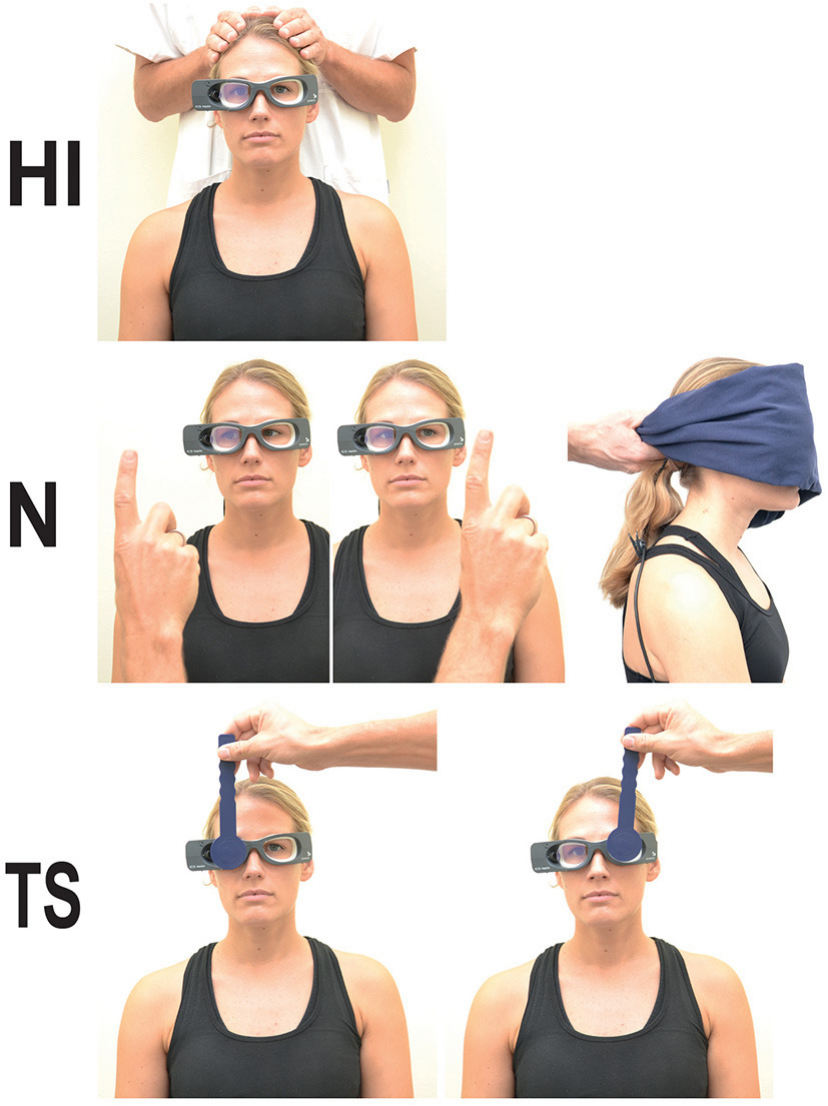
Performance of the three-step HINTS test battery: (HI) head impulse test, (N) nystagmus detection and (TS) test of skew. Nystagmus detection is performed in primary gaze position with and without fixation as well as looking about 20°–30° to the right and to the left.

As a first step, standard operating procedures for performance and interpretation of the TeleVertigo examination as well as for treatment options were implemented. A training program was set up for spoke staff members to perform vHIT, nystagmus detection and the alternating cover test (see Figure 1). This included 15 central training courses with 3.5 h of theoretical training and 2.5 h of practical training for doctors, nursing staff and therapists, on-site sessions for each hospital and also one-to-one online teachings per request. Furthermore, trained personnel were instructed to share knowledge with colleagues. All sessions were performed by the neuro-otologists and specialized physiotherapists of the two centers (Munich Clinic Harlaching and InnKlinikum Altötting). Teleneurologists in one hub hospital (Munich Clinic Harlaching) underwent individual training regarding the operation and interpretation of the acute examination. When necessary, supervision by a neuro-otologist was carried out during core working hours. At regular ongoing intervals, quality circles were organized for all participating hospitals.

### Technical Device

The additional TeleVertigo examination is performed using a VOG system (video goggles; ICS Impulse Type 1085 in combination with the OTOsuite Vestibular software, version 4.10 Build 1341, Natus Medical Denmark ApS, Taastrup, Denmark) as described elsewhere (19). These lightweight video goggles include a light, small and very fast infra-red camera, a half-silvered mirror reflecting infra-red light and small sensors measuring head movement velocity. Video goggles are secured to the patient's head with adjustable straps to minimize slippage. They were especially developed to document and quantify the vestibulo-ocular reflex (VOR). In the telemedical setting, those video goggles are used additionally as a second camera during videoconferencing for detection of nystagmus and skew deviation. This allows teleconsultants to explore even subtle eye movements, which is not possible with a standard camera. The video goggles are plugged into the videoconferencing system on demand. The teleconsultant operates the video goggle software and camera, and the doctor in the spoke hospital carries out examinations on the patient (e.g., giving head impulses, covering the eye, etc.).

### Hints Examination and Decision Rules

The telemedical HINTS examination was performed after a regular neurological examination and comprises oculography for nystagmus detection and test of skew as well as vHIT. According to the definition of the HINTS examination, we classified the result as peripheral vestibular lesion in case of direction fixed nystagmus, pathological vHIT and lack of skew deviation (each criterion must be met). A central vestibular lesion was assumed in case of direction-changing nystagmus and/or skew deviation or the combination of any nystagmus with a physiological vHIT. All cases without nystagmus were classified as unclear.

### Oculography for Nystagmus Detection

The patient is placed at 1 m distance from a fixation dot (on the wall). In order to perform an eye movement calibration of the VOG system, the patient is asked to stare at a projected dot. Gaze examination is performed to test for spontaneous and gaze evoked nystagmus. Therefore, the patient is instructed to look center, left and right (both about 20°-30°), first with fixation, then with fixation block. This latter is performed by covering the patient's eyes with an opaque towel. Nevertheless, the camera of the video goggles still enables the examination of eye movements. Each position is held for 15–30 s. Nystagmus analysis is carried out by the teleneurologist in the hub based on the evaluation of the video frame of the right eye. A direction changing gaze nystagmus strongly indicates a central lesion (22).

### Test of Skew Deviation

Skew deviation is tested through alternating cover test. The patient is instructed to stare at the dot on the wall again. The ED physician starts covering the right eye with one hand, then moves the hand to the left to cover the left eye, and back again to cover the right eye. Each eye is covered for about 5 s. This is repeated until each eye was covered at least five times. Through the camera the covered right eye is still visible to the teleneurologist. The teleneurologist evaluates whether there is a vertical adjustment movement of the right eye when covering or uncovering it. Skew deviation commonly indicates a vertebrobasilar pathology, especially brainstem strokes, but can also be caused by a peripheral vestibular pathology (9, 23, 24). A recent publication casts doubt on this interpretation (25).

### Video Head Impulse Test

During vHIT patients remain seated on the bed (or a stationary chair) at about 1 m distance from the dot on the wall which they are supposed to stare at. It is especially important that the straps of the video goggles are fixed tightly on the patient's head. The ED physician is standing behind the patient holding the patient's head with both hands above or below the straps. The ED physician rotates the patient's head horizontally at a small angle (about 10°-20°) in a random direction. In each direction (leftward and rightward) rotation is performed until 10–15 interpretable impulses are detected on both sides. Velocity of the stimulus (head movement) and velocity of the response (eye movement) are measured, displayed and processed by the OTOsuite software. Gain as ratio of eye movement velocity to head movement velocity is calculated. Individual gain of every single movement is displayed as well as the mean gain of all impulses on one side and shown on a diagram (gain vs. head velocity). Furthermore, head movement and eye movement velocity and possible catch-up saccades are displayed in a graph. Unilateral gain values <0.8 together with catch-up saccades are considered pathological, mostly indicating an unilateral vestibulopathy (16). A normal vHIT in the setting of AVS is a strong predictor of a central origin, mostly indicating stroke (22).

### Study Population

This study included consecutive patients who were admitted to EDs in the 11 participating primary care hospitals (TEMPiS stroke network spokes) and presented to the teleneurologist with acute dizziness, vertigo or imbalance of new quality within the last 72 h, without a new focal neurological deficit in the standard teleneurological examination and without any other obvious reason for the symptoms. Furthermore, fully trained staff in the spoke was mandatory, as well as fully trained teleneurologists in the hub. As only one teleneurologist team was trained, only those weeks in which the hub hospital in Munich Clinic Harlaching was on duty were included. Patients who had paroxysmal symptoms or completely resolved symptoms at time of consultation were included as well. Exclusion criteria were the unavailability of the examination *via* VOG system in the ED.

### Study Protocol

All variables were predefined. The following variables were collected prospectively from examinations stored in the software of the video goggles: vHIT parameters and duration of the examination. The other variables were retrieved from documentation of the telemedical consultations: age, sex, vascular risk factors, history of stroke/transient ischemic attack (TIA), peripheral vestibular disorder or dizziness/vertigo of unknown origin, symptoms which led to presentation in the emergency room, onset of dizziness/vertigo/imbalance, results of examination of nystagmus, skew deviation and side effects. Overall evaluation of telemedical HINTS examination (feasibility and categorization to peripheral or central cause), and reasons why tests were not feasible were documented. Primary outcome was the feasibility of the telemedical HINTS examination. Secondary outcomes were the feasibility and results of the single tests as well as categorization to peripheral or central cause. Additionally, we investigated why testing with video goggles was incomplete in some cases. All data were stored in an anonymized quality register, for which no patient consent is required according to German legislation.

### Statistical Analysis

As our primary and secondary outcomes of interest were descriptive in nature, we performed qualitative data analysis and used descriptive statistics.

## Results

**Figure 2:**
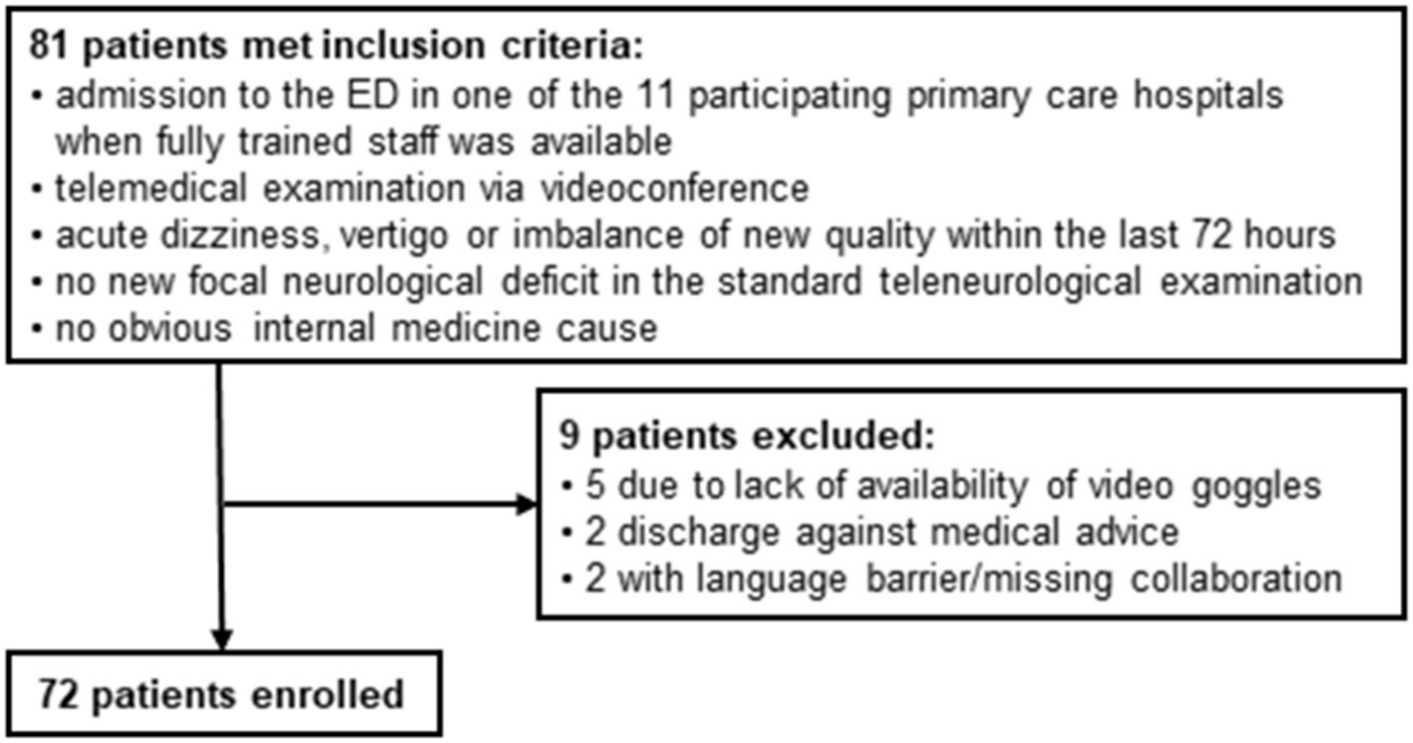
Study design.

From 01.06.2019 to 31.03.2020, 81 patients met the inclusion criteria for this study. 9 of them did not undergo an examination with the video goggle because of acute technical problems with the video goggles (*n* = 5), discharge against medical advice (*n* = 2) or language barrier with missing collaboration of the patient (*n* = 2) (see Figure 2).

In the remaining 72 cases an examination with the video goggles was performed. More female patients (*n* = 49; 68.1%) than male (*n* = 23; 31.9%) were included; mean age was 64.9 years. Median duration of dizziness/vertigo symptoms was 313 (IQR 231–653) minutes with almost half of the patients suffering from nausea (*n* = 32; 44.4%). For detailed baseline characteristics see Table 1. The median examination time for the telemedical HINTS examination and positional testing by Dix-Hallpike maneuver (results on that test are not reported in this manuscript) was 10 (IQR 6–15) minutes.

**Table 1:** Baseline characteristics of all patients enrolled.

	***n* = 72**
Age, years (mean, SD)	64.9 (15.4)
Sex	
Male	23 (31.9%)
Female	49 (68.1%)
Duration since onset of symptoms, minutes (median, IQR)	313 (231–653)
Symptoms	
Gait instability	23 (31.9%)
Nausea	32 (44.4%)
Oscillopsia	3 (4.2%)
Vascular risk factors	
Hypertension	40 (55.6%)
Hypercholesterinemia	5 (6.9%)
Diabetes	8 (11.1%)
Current/former smoking	4 (5.6%)
History of	
Previous peripheral vestibular disease	4 (5.6%)
Previous vertigo/dizziness of unknown origin	7 (9.7%)
Previous stroke/TIA	13 (18.1%)
Atrial fibrillation	4 (5.6%)
Coronary artery disease	8 (11.1%)

*IQR, interquartile range; SD, standard deviation*.

Successful evaluation of all three steps of the telemedical HINTS examination was feasible in 46 patients (63.9%) (Table 2). An investigation for nystagmus could be carried out in all patients (*n* = 72; 100%). The alternating covert test for search of skew was feasible in 70 (97.2%) and the vHIT in 47 (65.3%) patients. Mean peak velocity of head impulses was 213±37°/s on the right side and 209 ± 39°/s on the left side. Side effects, e.g., nausea and vomiting, during the examination occurred in 5 patients (6.9%).

**Table 2:** Intended and completed telemedical HINTS examinations.

	**Intended examinations**	**Examinations fully completed with interpretable results**
vHIT	72	47 (65.3%)
Nystagmus	72	72 (100%)
Test of skew	72	70 (97.2%)
**HINTS overall**	**72**	**46 (63.9%)**

*vHIT, video head impulse test; HINTS, Head Impulse Test, Nystagmus, Test of Skew*.

In two patients, the alternating cover test was not possible due to lack of cooperation. vHIT could not be carried out successfully in 25 patients mainly due to insufficient generation of evaluable impulses (*n* = 18; 72%). Further reasons were vomiting, acute technical problems and the lack of patient cooperation.

In 46 patients, all parts of the telemedical HINTS examination were performed successfully. In 21 of those cases (45.6%) a conclusive result could be determined by application of the HINTS rule. A peripheral vestibular lesion was identified in 7 cases (15.2%) with an abnormal gain (mean gain 0.45 ± 0.18) and correcting saccades (mean number of overt saccades 0.73 ± 0.13, latency 257 ± 61 ms, amplitude 213 ± 62°/s; mean number of covert saccades 0.20 ± 0.15, latency 119 ± 23 ms, amplitude 151 ± 92°/s) on one side and a normal gain on the other side (mean gain 0.93 ± 0.15). Peak velocity of head impulses in these 7 patients was 210 ± 35°/s on the right side and 209 ± 50°/s on the left side. A central vestibular lesion was diagnosed in 14 cases (30.4%) due to direction changing nystagmus in 4 cases (8.7%) and direction-fixed nystagmus in combination with normal vHIT in 10 cases (21.7%). Peak velocity of head impulses in these 14 cases was 220 ± 42 on the right side and 204 ± 31 on the left side with a mean gain of 1.02 ± 0.13 and 0.96 ± 0.15, respectively. In 25 cases (54.3%) the HINTS rule was not applicable as no nystagmus was detectable. For the application of the HINTS rule a nystagmus as part of a vestibular syndrome is required. Therefore, we classified these cases without any nystagmus as unclear with respect to the underlying etiology. For details see Table 3.

**Table 3:** Results of successful telemedical HINTS examinations (*n* = 46).

**vHIT**	**Nystagmus**	**Skew**	**Number**	**Interpretation**
**Pathological**	Direction-fixed nystagmus	No	7 (15.2%)	Peripheral cause
**Physiological**	Direction-fixed nystagmus	No	10 (21.7%)	Central cause
**Physiological**	Direction-changing nystagmus	No	4 (8.7%)	Central cause
**Physiological**	No nystagmus	No	25 (54.3%)	Unclear (HINTS not applicable due to missing nystagmus)

*vHIT, video head impulse test; HINTS, Head Impulse Test, Nystagmus, Test of Skew*.

## Discussion

Our prospective evaluation shows that telemedical HINTS examination can be performed successfully by trained staff *via* telemedicine in a relevant proportion of 63.9%. This study is the first evaluating a telemedical HINTS examination in ED patients of primary care hospitals. Our findings suggest that extensive area-covering and an 24/7 availability approach for the adequate workup of patients with acute dizziness and vertigo in hospitals without specialized neuro-otological care is possible. It may lead to a faster classification of these highly prevalent symptoms and may therefore be a relief not only for the patients but also regarding the socioeconomic burden. Reports are available on management improvements concerning dizzy patients *via* telemedicine. Zee et al. describe first experiences in the classification of patients with dizziness and vertigo in the ED by a “Tele-Dizzy consultation service” (26). Greater diagnostic accuracy *via* a telemedical connection to the neuro-otological department in the same academic center is reported. Similarly to our project, a VOG device was employed. All other published reports about telemedicine in dizzy/vertiginous patients focus on aspects others than the acute management in the emergency department (27–33).

Most challenging to perform but the single best predictor of a stroke is the vHIT. The full interpretability of almost two thirds of telemedical vHIT examinations in our cohort is therefore a remarkable result. Furthermore, based on the analysis of peak velocities of telemedical vHITs, peak velocities of head impulses of around 210°/s were reached, which is within recommended ranges (15). Nystagmus detection as well as test of skew were evaluated by clinical assessment of the transmitted video. The employed VOG system also allows a quantitative evaluation of these tests and may further improve diagnostic accuracy.

The time delay for an additional telemedical HINTS examination was <10 mins on average. This delay seems adequate for optimized triage with these symptoms.

In cases with successful telemedical HINTS examination, 46% could be classified as peripheral or central, leaving 54% of cases without classification according to the HINTS rule. Absence of a nystagmus in all those cases, made the HINTS rule non-applicable, and criteria for AVS were not met. AVS is a clinical syndrome of severe vertigo, nausea and vomiting, spontaneous nystagmus and postural instability (34). Recent studies estimate a proportion of AVS in all ED cases with acute dizziness/vertigo of about 10–20% (7). The higher proportion in our sample of 46% may be explained by case selection, as we did not include cases with obvious internal medicine cause, focal neurological deficit (explored in the teleneurological examination) and recurrent vertigo/dizziness symptoms of known quality. Nevertheless, with 54% of unclassifiable cases, the telemedical HINTS examination alone is of limited value for ED triage when criteria for AVS are not met. This is in line with recent publications (18). The addition of further symptom characteristics and tests may be of value. Further extensions of HINTS or new approaches to dizzy/vertiginous patients include addition of a bedside hearing test to HINTS (HINTS plus) to differentiate posterior fossa stroke from peripheral origin of vertigo (35), a more extended access including symptom timing, triggers and bedside eye examination (TiTrATE) (36) or adding standing examination and search for positional vertigo (STANDING) (37). Most of these items are integrated in the algorithm suggested by Venhovens et al. (38). Improved approaches to vertigo and dizziness with well-defined algorithms and further developed technology may offer new telemedical approaches (19, 39).

The most common reason in our study for not interpretable vHIT was the fact that not enough evaluable impulses could be generated. This difficulty may be overcome by more training and experience.

Twice as many cases suggesting a central lesion as cases with peripheral lesions (14 vs. 7) have been recorded in our sample when the telemedical HINTS examination was successful. Of note, among unsuccessful telemedical HINTS examinations in our sample, another 17 cases displayed direction-fixed nystagmus while vHIT was not interpretable. Most of these cases might have had a peripheral etiology, which, if identified, could have led to proportionally fewer central lesions in our findings. Once again, the rate of central lesions is higher than expected based on the available literature (6) and may be explained, in particular, by the generally more restrictive case selection criteria of our sample.

*Strengths* of our study are that it is a prospective and real-world analysis with data collection in multiple network spokes. This made it possible to collect a relevant number of consecutive patients (*n* = 81) over a 10-month-period. The disadvantage of a multicenter study concerning data collection and data interpretation was compensated in part by a central evaluation in one single center (Munich Clinic Harlaching).

There are some *limitations* to our study. First, telemedical HINTS examinations were not assessed by one single neuro-otologist, but we ensured an intensive training of all the involved teleneurologists. Furthermore, we cannot exclude some convenience sampling as it is just within the discretion of the treating ED physician of the spoke hospital to decide which cases are presented to the teleconsultation service and therefore included in the study.

To summarize, we showed that the telemedical HINTS examination is feasible within a telestroke network if close cooperation as well as intensive and ongoing training are established, and if continuous telemedical supervision by a neuro-otologist is available. The telemedical application of oculomotor tests in primary care hospitals offers the opportunity to improve acute hospital care of patients with acute dizziness and vertigo. For a full evaluation of the efficacy of this approach further studies are needed.

## Data Availability Statement

The raw data supporting the conclusions of this article will be made available by the authors, without undue reservation.

## Ethics Statement

Ethical review and approval was not required for the study on human participants in accordance with the local legislation and institutional requirements. Written informed consent for participation was not required for this study in accordance with the national legislation and the institutional requirements. Written informed consent was obtained from the individual(s) for the publication of any potentially identifiable images or data included in this article.

## Author Contributions

RM, CL, PM-B, NH, and GH contributed to conception and design of the study. RM, CL, and PM-B organized the database and performed the statistical analysis. RM and CL wrote the first draft of the manuscript. All authors contributed to manuscript revision, read, and approved the submitted version.

## Funding

CL and PM-B's efforts were supported by a grant from the Bavarian Ministry of Health and the German Foundation for Neurology (DSN).

## Conflict of Interest

HR is beta-tester of the ICS Impulse^®^ VOG system but has no financial interest in the product. He received honorary from Natus Medical ApS, Denmark, and Henning-Arzneimittel, Germany. PM-B and CL report grants from the Bavarian Ministry of Health and the German Foundation for Neurology (DSN) during the conduct of the study. The remaining authors declare that the research was conducted in the absence of any commercial or financial relationships that could be construed as a potential conflict of interest.

## Publisher's Note

All claims expressed in this article are solely those of the authors and do not necessarily represent those of their affiliated organizations, or those of the publisher, the editors and the reviewers. Any product that may be evaluated in this article, or claim that may be made by its manufacturer, is not guaranteed or endorsed by the publisher.

## Abbreviations

AVS: acute vestibular syndrome
CT: computed tomography
ED: emergency department
HINTS, Head Impulse Test, Nystagmus: Test of Skew
HIT, head impulse test, IQR: inter-quartile range
MRI: magnetic resonance imaging
ms: millisecond
NIHSS: National Institute of Health Stroke Scale
SD: standard deviation
TEMPiS: Telemedic Project for Integrative Stroke Care
TIA: transient ischemic attack
vHIT: video head impulse test
VOG: videooculography
VOR: vestibulo-ocular reflex.
